# Supplementary material for: Genomic Approaches to Identify Molecular Bases of Crop Resistance to Diseases and to Develop Future Breeding Strategies
Source: Int J Mol Sci. 2021 May 21;22(11):5423. doi: 10.3390/ijms22115423 (PMC8196592; doi:10.3390/ijms22115423)
Supplement: Supplementary file 1 [file ijms-22-05423-s001.zip › ijms-1176411-supplementary.pdf]

Table S1 Additional examples of use of genome editing technologies in resistance breeding of crop plants.

**Pathogen**	**Organism**	**Target S-gene**	**System and modification**	**Gene function / encoded protein**	**TF**	**Effect/ Phenotype**	**References**
**Bacterial**	Rice	*OsSWEET11*, *OsSWEET13*, *OsSWEET14* / promoter	CRISPR/Cas9 (Promoter disruption- Indels in promoter)	SWEET Sugar transporter Susceptibility to *X. oryzae*	Not	Enhanced resistance against BB disease	[1-2]
	Tomato	*SlDMR6*(downy mildew resistance 6)/cds region	CRISPR/Cas9 (Gene disruption -Indels in Exon 2 and 3)	2-oxoglutarate (2OG) Fe (II) -dependent oxygenases involved in hydroxylation or desaturation steps in plant hormone synthetic pathways, specifically up-regulated during pathogen infection. Susceptibility to *Pseudomonas syringae, P. capsici*, *Xanthomonas* spp.	Not	Increased salicylic acid levels. Enhanced broad spectrum resistance against bacterial speck, bacterial spot, *Phytophtora* blight	[3]
**Fungus**	Wheat	*TaEDR1* homeologous alleles / /cds region	CRISPR/Cas9 (Gene disruption - Indels in exon 4)	Raf-like mitogen activated protein kinase kinase kinase (MAPKKK) with a negative role in PM disease resistance Susceptibility to *Erysiphe cichoracearum*	Not	Enhanced tolerance to PM	[4]
	Rice	*OsERF922*/cds region	CRISPR/Cas9 (Gene disruption - Indels in target)	ERF family transcription factor implied in regulation of ethylene pathway. Susceptibility to Magnaporthe. oryzae	Yes	Enhanced resistance to rice blast	[5]
	Tomato	*PMR4*/cds region	CRISPR/Cas9 (Deletion and inversion in target)	PMR4 encodes for a callose synthase that is responsible for the production of callose in response to biotic and abiotic stresses. Susceptibility to *O. neolycopersici*	Not	Enhanced resistance to tomato PM	[6]
	Tomato	*PMR4*/cds region	CRISPR/Cas9 (Gene disruption - Indels in target)	Susceptibility to *O. neolycopersici*	Not	Reduced but not complete loss of susceptibility to the PM pathogen	[7]

	Tomato	*Solyc08g075770* (Ortholog of *SlDMR6-1)*/cds region	CRISPR/Cas9 (Gene disruption - Indels in target)	Transmembrane protein involved in regulation of ROS accumulation and SA and JA defense signaling pathways. Susceptibility to *Fusarium oxysporum* f. sp. *lycopersici*	Not	Enhanced susceptibility to fusarium wilt	[8]
	Papaya	*PpalEPIC8*/cds region	CRISPR/Cas9 (Gene disruption- Deletion in target)	Susceptibility to *Phytophthora palmivora*	N.D.	Enhanced resistance against *P. palmivora*	[9]
**Bacterial and fungus**	Rice	*TMS/Pi21/Xa13**/ cds region	CRISPR/Cas9 multiplex genome editing system (Gene disruption - Indels in target)	Susceptibility to *M. oryzae* and *X. oryzae* *esempio*	Yes	Homozygous triple *tms5/pi21/xa13* mutants in the T1generation with characteristics of thermo-sensitive genic male sterility (TGMS) and enhanced resistance to rice blast and bacterial blight.	[10]
**Viruses - Viral genome** <br>***DNA viral disease***	Tobacco	*Rep*	TALEN (mutation n.d.)	Susceptibility to TbCSV, TYCCNV and TLCYnV	Not	Partial disease resistance (DNA viral disease)	[11]
	Tobacco	*IR and C1*	CRISPR/Cas9 Indels	Susceptibility to CLCuMuV	Not	Complete resistance to CLCuMuV (DNA viral disease)	[12]
**Viruses - Viral genome** <br>***RNA viral disease***	Potato	*P3, CI, NIb* and *C P* conserved regions of *PVY* genomes	CRISPR/Cas13a	Susceptibility to multiple strains of Potato Virus Y (PVY)	Not	Suppressed PVY accumulation and disease symptoms	[13]
**Viruses - Host factors**	Tomato	*DCL2b*/cds region	CRISPR/Cas9 Indels	Roles in small RNA biogenesis and in antiviral defense. Susceptibility to PVX, TMV	Not	Mutants showed viral symptoms when injected by targeted viruses (RNA viral disease)	[14-15]

TF= Transgene free; ND: not determined
Virus: TMV, tobacco mosaic virus; PVX, potato virus X;
Genes: CP, coat protein; Rep, replication association protein; IR, intergenic region; Pi21 encodes a proline-rich protein for rice blast resistance; Xa13= sugar transporter sweet11; TMS= thermo-sensitive male sterility.

References

1. Zhou, H.; Liu, B.; Weeks, D.P.; Spalding, M.H.; Yang, B. Large Chromosomal Deletions and Heritable Small Genetic Changes Induced by CRISPR in Rice. Nucleic Acids Res. 2014, 42, 10903–10914.
2. Zhou, J.; Peng, Z.; Long, J.; Sosso, D.; Liu, B.; Eom, J.S.; Huang, S.; Liu, S.; Vera Cruz, C.; Frommer, W.B.; et al. Gene Targeting by the TAL Effector PthXo2 Reveals Cryptic Resistance Gene for Bacterial Blight of Rice. Plant J. 2015, 82, 632–643.
3. Deng, Z.; Wang, Q.; Liu, Z.; Zhang, M.; Machado, A.C.D.; Chiu, T.P.; Feng, C.; Zhang, Q.; Yu, L.; Qi, L.; et al. Mechanistic Insights into Metal Ion Activation and Operator Recognition by the Ferric Uptake Regulator. Nat. Commun. 2015, 6, 7642.
4. Zhang, Y.; Zhen, L.; Yuan, Z.; Yanpeng, W.; Jinxing, L.; Kunling, C.; Jin-Long, Q.; Caixia, G. Efficient and Transgene-free Genome Editing in Wheat through Transient Expression of CRISPR/Cas9 DNA or RNA. Nat. Commun. 2017, 7, 12617.
5. Wang, F.; Wang, C.; Liu, P.; Lei, C.; Hao, W.; Gao, Y.; Liu, Y.G.; Zhao, K. Enhanced Rice Blast Resistance by CRISPR/Cas9-targeted Mutagenesis of the ERF Transcription Factor Gene OsERF922. PLoS ONE 2016, 11, e0154027.
6. Koseoglou, E. The Study of SlPMR4 CRISPR/Cas9-Mediated Tomato Allelic Series for Resistance Against Powdery Mildew. Masters’s Thesis, Department of Plant Breeding Wageningen University and Research, Wageningen, The Netherlands, 2017.
7. Santillán Martínez, M.I.; Bracuto, V.; Koseoglou, E.; Appiano, M.; Jacobsen, E.; Visser, R.G.F.; Wolters, A.A.; Bai, Y. CRISPR/Cas9-targeted Mutagenesis of the Tomato Susceptibility Gene PMR4 for Resistance against Powdery Mildew. BMC Plant Biol. 2020, 20, 284.
8. Prihatna, C.; Barbetti, M.; Barker, S. A Novel Tomato Fusarium Wilt Tolerance Gene. Front. Microbiol. 2018, 9, 1226.
9. Gumtow, R.; Wu, D.; Uchida, J.; Tian, M. A Phytophthora Palmivora Extracellular Cystatin-like Protease Inhibitor Targets Papain to Contribute to Virulence on Papaya. Mol. Plant Microbe Interact. 2018, 31, 363–373.
10. Li, S.; Shen, L.; Hu, P.; Liu, Q.; Zhu, X.; Qian, Q, Wang, K.; Wang, Y. Developing Disease-resistant Thermosensitive Male Sterile Rice by Multiplex Gene Editing. J. Integr. Plant Biol. 2019, 61, 1201–1205.
11. Cheng, X.; Li, F.; Cai, J.; Chen, W.; Zhao, N.; Sun, Y.; Guo, Y.; Yang, X.; Wu, X. Artificial TALE as a Convenient Protein Platform for Engineering Broad-spectrum Resistance to Begomoviruses. Viruses 2015, 7, 4772–4782.
12. Yin, K.; Han, T.; Xie, K.; Zhao, J.; Song, J.; Liu, Y. Engineer Complete Resistance to Cotton Leaf Curl Multan Virus by the CRISPR/Cas9 System in Nicotiana benthamiana. Phytopathol. Res. 2019, 1, 9.
13. Zhan, X.; Zhang, F.; Zhong, Z.; Chen, R.; Wang, Y.; Chang, L.; Bock, R.; Bihua, N.; Zhang, J. Generation of Virus-resistant Potato Plants by RNA Genome Targeting. Plant Biotechnol. J. 2019, 17, 1814–1822.
14. Wang, T.; Deng, Z.; Zhang, X.; Wang, H.; Wang, Y.; Liu, X.; Liu, S.; Xu, F.; Li, T.; Fu, D.; et al. Tomato DCL2b is Required for the Biosynthesis of 22-nt Small RNAs, the Resulting Secondary siRNAs, and the Host Defense against ToMV. Hortic. Res. 2018, 5, 62.
15. Wang, Z.; Hardcastle, T.J.; Pastor, A.C.; Yip, W.H.; Tang, S.; Baulcombe, D.C. A Novel DCL2-dependent miRNA Pathway in Tomato Affects Susceptibility to RNA Viruses. Genes Dev. 2018, 32, 1155–1160
